# Unlocking the potential of uncontrolled DCD in lung transplantation: A review of 2 decades of experience

**DOI:** 10.1016/j.jhlto.2025.100374

**Published:** 2025-08-14

**Authors:** Irene Bello, Alessandro Palleschi, Marcelo Cypel, Eduard Argudo, Alberto Sandiumenge

**Affiliations:** a Thoracic Surgery Department, Vall d′Hebron University Hospital, Barcelona, Spain; b Donation and Transplantation of Organs, Tissues and Cells, VHIR, Barcelona, Spain; c Department of Pathophysiology and Transplantation, University of Milan, Milan, Italy; d Thoracic Surgery and Lung Transplantation Unit, Fondazione IRCCS Ca' Granda - Ospedale Maggiore Policlinico, Milan, Italy; e Toronto Lung Transplant Program, University of Toronto, Toronto, Canada; f Intensive Care Medicine Department, Vall d′Hebron University Hospital, Barcelona, Spain; g Shock, Organ Disfunction and Resuscitation Research Group (SODIR), Vall d′Hebron Research Institute (VHIR), Barcelona, Spain; h Department of Donor & Transplant Coordination, Vall d′Hebron University Hospital, Barcelona, Spain

**Keywords:** uncontrolled donation after circulatory death, lung transplantation, lung donor, uncontrolled donor after cardiac death, exvivo lung perfusion

## Abstract

Uncontrolled donation after circulatory death (uDCD) represents a promising yet underutilized approach to expanding the lung donor pool amid persistent organ shortages. Since the first successful lung transplantation from a uDCD donor in 2001, increasing clinical experience and advancements in organ preservation have demonstrated its feasibility. This review critically explores historical evolution, physiological basis, preservation techniques, ethical and legal considerations, and clinical outcomes of uDCD lung transplantation. The lung's unique ability to maintain viability through passive oxygen diffusion in the absence of perfusion supports its potential in the uDCD context. Compared to donors after brain death (DBD), uDCD donors may avoid systemic inflammatory response, potentially preserving graft quality. However, concerns persist regarding ischemia-reperfusion injury and mitochondrial dysfunction, highlighting the need for mitigation strategies such as ex vivo lung perfusion and normothermic ventilation. Ethical and legal challenges—particularly those related to the determination of death and consent—remain key obstacles. Organizational demands, including rapid coordination between prehospital, hospital teams and transplant teams, further limit broader implementation. Despite these barriers, reported outcomes are encouraging: to date, over 70 transplants from uDCD donors have been documented, with 1-year survival rates ranging from 71% to 87.5% and long-term outcomes comparable to DBD transplants. Integration of uDCD into routine clinical practice will require standardized protocols, robust public engagement, and institutional commitment. When appropriately implemented, uDCD lung transplantation offers a viable opportunity to increase donor availability and improve access to life-saving treatment.

## Background

Lung transplantation (LTx) remains a critical treatment for patients with end-stage lung disease. The number of lung transplants increased by 15% in 2024 compared to 2022. However, the persistent shortage and underutilization of lung donors remain a significant obstacle. To mitigate this issue, several approaches have been adopted, including enhanced identification of donors in intensive care units (ICUs),^1^, ^2^ ex vivo lung perfusion (EVLP) or the implementation of approaches such as DCD, including both controlled and uncontrolled donors. While controlled DCD is increasingly accepted and integrated into routine practice in several countries, uncontrolled DCD remains considerably more complex due to unpredictable timing, logistical constraints, and ethical implications.

Since the first on porpoise LTx from uDCD was reported by Steen et al,^3^ this technique has gained acceptance in several countries, encouraging clinical outcomes.^4^ Nevertheless, disparities persist across nations due to variations in legal frameworks, logistical challenges, and organizational structures.

This review critically evaluates the current state of uDCD LTx, focusing on donor management, preservation techniques, ethical implications, and clinical outcomes. The aim is to provide insights into the challenges and opportunities of integrating uDCD into standard LTx practices and to serve as a foundation for future research and clinical application.

## History

### Classification and terminology

In Europe, the term non-heart-beating donor was historically used to describe an organ donor after cardio-respiratory arrest. This terminology originated from the Maastricht classification, which was developed during a workshop held in Maastricht in 1995.^5^ The classification arose from the need to distinguish between different categories of potential donors in various end-of-life scenarios (Table 1).

**Table 1:** The Maastricht Categories

Category I	Dead on arrival at hospital
Category II	Death with unsuccessful resuscitation
Category III	Awaiting cardiac death
Category IV	Cardiac arrest while brain dead

The term non-heart-beating donor caused confusion regarding the definition of death, as it was perceived to be based on the failure of a single organ rather than the whole patient. Thus, the Institute of Medicine—American National Academy of Sciences^6^ proposed renaming it Donation after Circulatory death, a concept later adopted by the World Health Organization.^7^ In 2011, the classification was modified in Madrid and in the Eurotransplant^8^ zone to better reflect the realities of these countries. During the DCD Conference in Paris in 2013, it was agreed to modify the original Maastricht classification and update it with the new developments. An additional category was added to account for countries—such as Belgium, the Netherlands, Luxembourg, Canada, and more recently Spain—that formally recognize organ donation after euthanasia/medical assistance in dying. This type was described as the fifth category^9^ (Table 2).

**Table 2:** The Maastricht Categories Readjusted by Erasmus et al^10^

Uncontrolled DCD	I	Dead in the out-of-hospital setting	1A. Cardiocirculatory death outside hospital with no witness. Totally uncontrolled
			1B. Cardiocirculatory death outside hospital with witnesses and rapid resuscitation attempt. Uncontrolled
	II	Unsuccessful resuscitation	2A. Unexpected cardiocirculatory death in ICU. Uncontrolled
			2B. Unexpected cardiocirculatory death in hospital (ER or ward), with witnesses and rapid resuscitation attempt. Uncontrolled
Controlled DCD	III	Awaiting cardiac arrest	3A. Expected cardiocirculatory death in ICU. Controlled
			3B. Expected cardiocirculatory death in OR (withdrawal phase > 30 min). Controlled
			3C. Expected cardiocirculatory death in OR (withdrawal phase < 30 min). (Highly) controlled
	IV	Cardiac arrest while brain death	4A. Unexpected cardiocirculatory arrest in a brain dead donor (in ICU). Uncontrolled
			4B. Expected cardiocirculatory arrest in a brain dead donor (in OR or ICU). (Highly) controlled
	V	Euthanasia	5A. Medically assisted cardiocirculatory death in ICU or ward. Controlled
			5B. Medically assisted cardiocirculatory death in OR. Highly controlled

Abbreviations: DCD, donor after cardiac death; ICU, intensive care unit; OR, operating room.

### uDCD lung donor

The first LTx in history was performed by Dr Hardy in 1963 using uDCD donor. The number of LTx from DCD increased until 1980, when the Uniform Determination of Death Act was approved in the United States. The law codified a unified approach to declare the death of a patient. Its interpretation sparked considerable debate, and with the establishment of brain death criteria, DCD donors were progressively abandoned in LTx protocols.

Thanks to improvements in preservation techniques, interest in DCD donors was renewed. In 2001, Steen published the first successful clinical LTx using uDCD IIB donor.^3^ The donor was a man who suffered a cardiac arrest with unsuccessful cardiopulmonary resuscitation (CPR). Sixty-five minutes post declaration of death, the next of kin gave permission to insert 2 chest drains and initiate topical cooling. The heart-lung block was harvested 3 hours later and perfused with EVLP and right lung was transplanted after it. The recipient recovered within 2 weeks.

In 2004, Nuñez Peña et al^11^ reported 2 LTx cases from uDCD IIA. Rodríguez et al^12^ showed the good outcomes of this type of donors. The results achieved by this group showed that uDCD lung donors could be a good.^12^ The Spanish technique included the insertion of a 24F chest drain into each hemithorax, infusion of cold Perfadex solution (Medisan, Uppsala, Sweden) at 4°C into the pleural space for topical cooling, and the cessation of mechanical ventilation. In vivo assessments were performed by infusing 300 ml of the donor’s blood through the pulmonary artery and conducting a temperature-corrected arterial blood gas analysis on the left atrium and each pulmonary veins.

This technique introduced logistical, ethical, and legal complexities in many countries; to address this, new strategies were developed to simplify the process.^13^ The Milan transplantation center^14^ published the first case of uDCD lung donor preserved using simple recruitment maneuvers (RMs), protective normothermic ventilation and evaluated by EVLP. Healey et al^15^ published the initial North American experiences with uDCD lung donors preserved via lung inflation implemented using a continuous positive airway pressure and EVLP assessment. Both of these techniques allowed the lungs to be preserved without aggressive maneuvres, like to insert chest-tubes, which could be in conflict with ethical and legal framework.

In 2011, Mulloy et al^16^ demonstrated in a swine model improved outcomes following EVLP reconditioning after uDCD donation. Erasmus et al^10^ recommended the ex-situ assessment with EVLP in uDCD lung donors. Tanaka et al^17^ also published their experience with this technique and subsequent EVLP evaluation to enhance the functional assessment of uDCD lungs. At present, EVLP is useful, not to say essential tool to evaluate uDCD. Campos-Cañaveral et al^4^ considered an EVLP platform essential to develop and maintain a uDCD program.

## Lung physiology

Lung physiology offers intrinsic advantages that may support the safer use of uDCD lung donors.

### Lung tissue oxygenation

The lung is a unique organ in that it does not rely solely on vascular perfusion for oxygenation but can instead receive oxygen through passive diffusion across the alveolar surface. Egan et al^18^ conducted LTx in an animal model after 2 hours of circulatory absence, achieving promising results. The critical factor was adequate lung inflation and high intra-alveolar oxygen concentration. Greco et al^19^ reproduced the experiment in a pig model with 30, 60, and 90 minutes of warm ischemia time (WIT), and they reported comparable results across all groups. Ulicny^20^ extended the WIT to 4 hours with continuous ventilation. Adequate gas exchange was demonstrated even after prolonged WIT in the absence of circulation, highlighting a key physiological advantage of the lung in the DCD setting.

### Inflammatory response

Although lung grafts may be exposed to warm ischemia and subsequent mitochondrial damage, uDCD donors do not undergo brain death and therefore avoid the associated inflammatory cascade. Brain death is followed immediately by a sympathetic storm, with increased levels of norepinephrine and epinephrine, and induces changes in the hypothalamic-pituitary axis, decreasing the levels of adrenocorticotrophic hormone, cortisol, triiodothyronine, thyroxine, insulin, and vasopressin.^21^ These changes cause initial hypertension and tachycardia. Subsequently, systemic arterial pressure decreases due to vasodilatation. This catecholamine surge, combined with endocrine and hemodynamic disturbances, leads to metabolic derangements, including reactive oxygen species (ROS) production, energy depletion, and lactic acidosis.^22^ Brain death induces a systemic inflammatory and immunologic response with increased serum levels of proinflammatory cytokines.^23^ Sandiumenge et al^24^ showed higher levels of IL-6, IL-8, and IL-10 in donors after brain death (DBD) donors compared to DCD.

Despite this, Van de Wauwer^25^ showed an increased concentration of catecholamine hormones donors dead by hypoxic cardiac arrest in a pig DCD model. They suggested that the agonal phase during hypoxia may trigger a catecholamine surge similar to that observed in DBD donors.

### Mitochondrial damage

During WIT, lungs experience oxygen deprivation at body temperature, leading to cellular injury that is exacerbated upon reperfusion. Ischemia disrupts mitochondrial homeostasis via calcium overload, oxidative stress, and impaired mitochondrial dynamics. While WIT is critical in organs such as the liver and heart, lungs appear to tolerate certain WIT durations without significant impairment.

One of the primary mechanisms involves the production of ROS during the ischemic phase. In lung donors, oxygen deprivation during warm ischemia halts oxidative phosphorylation, resulting in mitochondrial dysfunction and accumulation of reducing equivalents such as NADH and FADH₂.^26^ When oxygen is reintroduced (e.g., during reperfusion), these reducing equivalents are rapidly oxidized, overwhelming the electron transport chain. This causes electron leakage, primarily at complexes I and III of the electron transport chain, which reduces molecular oxygen to form superoxide, a key ROS precursor.^27^ This promotes oxidative damage to mitochondrial DNA (mtDNA) and disrupts respiratory chain complexes, reducing ATP synthesis. Moreover, ischemia activates xanthine oxidase, an enzyme that contributes to ROS production during hypoxia and reperfusion. Hypoxia leads to hypoxanthine accumulation, which, upon oxygen reintroduction, is metabolized by xanthine oxidase to generate superoxide and hydrogen peroxide.^28^

Another key mechanism involves the mitochondrial permeability transition pore (mPTP), which opens in response to calcium overload during ischemia, resulting in loss of mitochondrial membrane potential (ΔΨm) and cytochrome C release. This promotes apoptotic and necrotic cell death. Studies also indicate that ischemia induces ΔΨm hyperpolarization priming mitochondria for dysfunction during reperfusion.^29^ The sudden return of oxygen leads to a burst of ROS, oxidizing mitochondrial inner membrane components such as cardiolipin and protein thiols, increasing mPTP sensitivity.^29^ In addition, oxidative stress may trigger translocation of proapoptotic proteins like Bax and p53 to mitochondria, further destabilizing the membrane and promoting pore opening.

The protein p53 is a central stress sensor. Its translocation to mitochondria and complex formation with cyclophilin D promotes mPTP opening and necrosis.^30^ During ischemia, the mPTP remains closed and opens only upon reperfusion. During ischemia, the pore remains closed and opens only upon reperfusion. When it does, excessive Ca²⁺ influx can lead to mitochondrial swelling and rupture of the outer membrane.^31^

Mitochondrial injury also disrupts mtDNA. Damaged mtDNA and impaired respiratory function result in the release of mtDNA as damage-associated molecular patterns (DAMPs) interact with Toll-like receptor 9, activating inflammatory cascades and neutrophil extracellular trap formation, further damaging lung tissue.^32^ This local injury may propagate to distant organs. Sun et al^33^ identified a correlation between circulating DAMPs and increased endothelial permeability. Bello et al^34^ did not observe elevated DAMP concentrations in cDCD donors compared to DBD donors.

Differences in inflammatory response and mitochondrial injury impact lung tissue integrity. DCD and DBD donors exhibit distinct gene expression profiles.^35^ Baciu et al^36^ observed greater upregulation of inflammatory pathways in DBD, whereas cDCD samples showed transcriptomic signatures linked to cell death, apoptosis, and necrosis.

## Ethical, legal, and social issues

DCD, including uDCD, raises several legal, social, and ethical challenges that may hinder the implementation of such programs.

One of the most relevant ethical issues concerns how best to act in the patient’s interests when survival is no longer possible. There is a perceived conflict between maximizing the chances of successful resuscitation and ensuring the opportunity to donate viable organs.^37^ This conflict becomes even more evident with the use of extracorporeal cardiopulmonary resuscitation (ECPR), particularly in patient’s refractory to standard CPR. There may be concerns about whether physicians are forced to choose between pursuing organ donation or attempting CPR, potentially influencing the decision to transfer a patient to a center that offers ECPR with subsequent consideration of organ donation.^38^

For that reason, many authors propose clear role separation between those making decisions regarding resuscitation and those involved in enrolling patients into uDCD protocols. They also advocate initiating uDCD programs only in centers with well-established ECPR programs to maximize the potential for patient survival and/or organ donation in cases where resuscitation proven unsuccessful.^39^

Another ethical and legal constraint for uDCD programs lies in the definition of death based on circulatory criteria, which, in the context of organ donation, some authors argue should meet a higher standard of evidence than in nondonation scenarios.^40^ Debate regarding the declaration of death centers on concepts such as permanence (that circulation will not be restored), irreversibility (that circulation cannot be restored), and the duration of “hands-off-period” (the time between termination of resuscitation and declaration of death). However, some authors argue that declaring death in the uDCD setting is less complex, since the patient has already undergone rigorous and unsuccessful resuscitative efforts.^41^

Following the uDCD pathway, once death has been declared, early and efficient initiation of preservation techniques, such as vessel cannulation and organ perfusion, should be started. However, this raises ethical concerns, as it may carry the risk of inadvertently restoring cerebral circulation, thus potentially violating the “dead donor rule.” This is particularly relevant when using normothermic regional perfusion (NRP). The occlusion of arterial flow via an aortic occlusion balloon at the thoracic aorta can facilitate the preservation of abdominal organs while preventing perfusion of the brain.^42^ Approaching families or next of kin in the uDCD context often poses a challenge, as they are frequently under immense emotional stress during the impending loss of a loved one*.* When combined with the time constraints associated with this type of donation, concerns arise about their ability to provide (*appropriate*) informed consent. In all cases, families should be approached with sensitivity and respect by staff specifically trained.^43^

The implementation of uDCD programs requires substantial human, logistical, and economic resources because of the WIT, and preservation time, the number of futile activations and the high number of professionals needed, and it demands fine-tuned coordination between prehospital and hospital teams to optimize the results.^44^ However, several authors suggest that long-term costs may be offset by reducing the number of waiting list.^45^ On the other hand, organ yield and viability in uDCD programs remain lower than in cDCD and DBD programs. Moreover, although some studies suggest that organs transplanted from uDCD donors perform comparably to those from DBD or cDCD donors, other case series studies indicate a higher incidence of short-term and long-term complications such as delayed graft function or poorer survival.^46^, ^47^ For this reason, some authors advocate obtaining explicit recipient consent when such organs are used, to ensure transparency*.*

Public engagement and trust are of vital importance, especially relevant in the uDCD setting. Several authors reported that current public understanding of uDCD is poor as is the knowledge that many clinicians have on this type of donation.^48^

Public education about organ donation in general, and uDCD in particular, is essential. Equally important is training the medical community not only in technical aspects but also in the legal and ethical considerations surrounding donation*.* It has been demonstrated that increasing knowledge leads to a more positive perception and attitude toward these types of programs.^49^

## uDCD organization

uDCD programs exhibit considerable variability in their organizational structures, influenced by regional legislation, ethical considerations, and health care infrastructure. Although some uDCD programs are exclusively focused on lung donation, the majority are integrated within broader uDCD protocols that also aim to preserve and procure abdominal organs.^50^ Such protocols require a highly sophisticated and comprehensive organizational framework, starting from prehospital emergency medical services and extending into the hospital environment, where transplant coordinators and specialized medical teams are required to sustain life-support interventions such as vascular cannulation, initiate extracorporeal interval support for organ retrieval, implement NRP, or carry out dedicated lung preservation procedures.^51^

The process is typically initiated by the identification of potential donors by prehospital emergency medical services teams. Upon identification, the designated receiving center is promptly notified to activate the necessary logistical and clinical preparations. At the hospital, a specialized medical team assesses the patient to confirm the absence of viable therapeutic interventions, ensuring that all options for resuscitation or recovery have been exhausted. A legally mandated "no-touch" interval is then observed, the duration of which varies according to national regulations, to unequivocally establish the irreversible cessation of circulatory function. Following the formal declaration of death, organ preservation strategies are promptly initiated, which may include the reinitiation of chest compressions and mechanical ventilation, along with the implementation of extracorporeal interval support for organ retrieval or other targeted techniques depending on the organs intended for procurement.^52^

In countries where uDCD programs are well-established, such as Spain or Italy, the growing use of ECMO for refractory cardiac arrest has raised significant ethical considerations, due to the substantial logistical and organizational overlap between ECPR and uDCD pathways.^53^, ^54^ In response, several groups have developed integrated programs that encompass both strategies.^55^, ^56^ Integrated ECPR and uDCD programs are designed to first ensure that all therapeutic options are exhausted for potentially viable patients by providing advanced circulatory support through ECPR. This initial approach not only offers a chance of survival but also preserves the possibility of organ donation through brain death or controlled DCD if neurological recovery is not achieved.^57^, ^58^ For patients deemed nonsurvivable despite ECPR, these programs enable a seamless transition to uDCD protocols, maintaining the opportunity for organ donation in accordance with the patient’s or family’s wishes (Table 3).^55^, ^56^

**Table 3:** Clinical Indications and Differences: ECPR Versus uDCD

Characteristic	ECPR	uDCD
Clinical scenario	Refractory cardiac arrest with potential for recovery.	Cardiac arrest with no chance of recovery, after failed resuscitation.
Patient eligibility	Witnessed cardiac arrest, initial shockable rhythm preferred, short no-flow and low-flow times, absence of severe comorbidities.	Witnessed cardiac arrest in whom resuscitation efforts have failed, no return of spontaneous circulation, and death has been declared.
Primary goal	Attempt to restore circulation and neurological function.	Preserve organ viability after death for transplantation purposes.
Timing	As early as possible after cardiac arrest, ideally within minutes of recognizing refractory arrest.	Initiated after declaration of death and observation of the no-touch period.
Low-flow time limit	Aims for <60 min	<150 min
Logistics	Requires immediate ECMO team availability, cannulation on scene or upon hospital arrival.	Requires ECMO team availability, coordination with transplant team, and initiation of preservation protocols.
Organ donation outcome	Possible, if the patient progresses to brain death or cDCD after bad neurological outcome	Direct progression to organ procurement, primarily kidneys and lungs, sometimes liver or pancreas.
Ethical focus	Prioritization of life-saving intervention and potential for survival.	Respect for donor autonomy and preservation of organ viability for transplantation.

Abbreviations: ECPR, extracorporeal cardiopulmonary resuscitation; uDCD, uncontrolled donation after circulatory death.

## uDCD models

Lungs are the only organs that do not require circulation to maintain aerobic cellular metabolism.

## Hypothermic-non ventilated technique

**Image 1:**
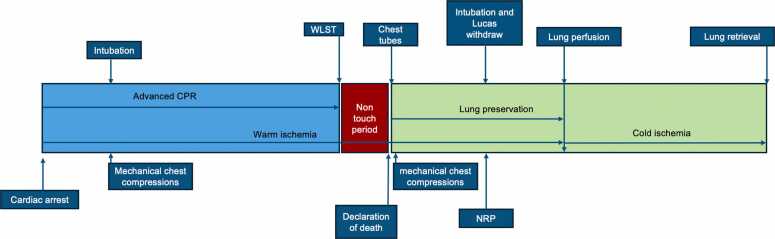
Non ventilation lungs with topical cooling technique. CPR, cardiopulmonary resuscitation; WLST, withdraw support treatment.

The first uDCD lung transplant was performed in 2001 by Dr Steen.^3^ The preservation technique consisted of topical cooling via the insertion of 2 chest drains, without the use of ventilation. This technique was subsequently reproduced by the team at Hospital Puerta de Hierro in Madrid, Spain, starting in 2004 (Image 1).

Following a witnessed cardiac arrest, out-of-hospital emergency units initiate basic and advanced CPR maneuvers within 15 minutes of receiving the emergency call. These interventions continue during transport to the Emergency Department until death is confirmed. The transfer of a potential donor requires maintaining cardiac compressions using mechanical devices, along with mechanical ventilation. These devices do not cause lung injury that would render lungs unsuitable for transplantation.^59^, ^60^

Death is declared in the hospital by a professional independent from the resuscitation team. This is done according to the following criteria^52^:

− Unsuccessful CPR in accordance with national protocols aligned with international standards.
− Cessation of circulation and respiration confirmed by absence of electrical activity.
− A legally defined minimum observation period ("no-touch period"), ranging from 5 to 20 minutes depending on the country

After death is determined and certified, legal authorization for organ preservation is requested from the on-duty judge. Cardiac compression and mechanical ventilation are restarted. Serologic tests, blood samples, and a chest X-ray are performed. The donor is administered intravenous heparin (3-5 mg/kg) to prevent coagulation, and an extracorporeal membrane oxygenation (ECMO) system is connected via the femoral vein and artery for abdominal organ preservation. To prevent the abdominal preservation solution and potential toxins from entering the cardiopulmonary circulation, a Fogarty catheter is inserted into the contralateral femoral artery and inflated at the supradiaphragmatic level.

After initiation of abdominal organ preservation, cardiac compression and mechanical ventilation are discontinued. Bilateral thoracic drains are inserted at the second intercostal space along the mid-clavicular line. A cold preservation solution, such as Perfadex, chilled to 4°C, is infused (5-6 liters per hemithorax) to induce topical cooling and facilitate lung collapse. The esophageal temperature is maintained at approximately 20°C during the procedure. When abdominal preservation using NRP is performed, or to enhance lung cooling, some teams employ a recirculation system for the lung preservation solution to sustain the target temperature. For this purpose, 2 additional thoracic drains are inserted at the sixth intercostal space along the mid-axillary line.

Before the procedure, approximately 300 ml of venous blood is collected from the potential donor and stored at 4°C for later functional lung evaluation. Next-of-kin consent is then obtained, followed by formal authorization for organ procurement. Maximum preservation time varies between centers but is generally limited to 240 minutes.^61^, ^62^

Following informed consent, the topical cooling preservation solution is evacuated from both pleural cavities. Mechanical ventilation is initiated using 100% fractional inspired oxygen (FiO₂) and a positive end-expiratory pressure of 5 cm H₂O. A bronchoscopy examination is subsequently conducted to exclude the presence of gastric aspiration.

Antegrade perfusion of the lungs is carried out via the pulmonary artery using 5 to 6 liters of Perfadex solution, continued until the effluent from the left atrium runs clear. Then, 300 ml of donor’s blood is infused into the pulmonary artery, followed by arterial blood gas analysis of the effluent from the left atrium (corrected for temperature) and all main pulmonary veins. Perfusion is typically conducted at a controlled temperature range of 4°C to 10°C. The procedure concludes with retrograde Perfadex perfusion through the pulmonary veins.

The lungs are then surgically procured and stored under appropriate preservation conditions.

### Normothermic-oxygenated technique

**Image 2:**
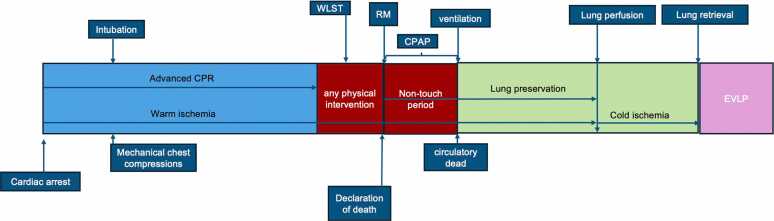
Normothermic-oxygenated technique. CPR, cardiopulmonary resuscitation; EVLP, ex vivo lung perfusion; RM, recruitment maneuver; WLST, withdraw support treatment.

After some preclinical studies, such as those by Sakamoto et al,^63^ who demonstrated that ventilation after cardiac arrest reduces ischemia-reperfusion injury, Valenza et al^14^ reported the first clinical protocol for lung procurement in uDCD using a ventilation-only strategy without topical cooling, followed by EVLP reconditioning, with excellent outcomes (Image 2). A novel technique proposed in an animal model by Junior et al^64^ combined ventilation with topical cooling and sevoflurane inhaled postmortem with promising results.

After cardiovascular collapse, patients are treated by an advanced life support team on-site and then transported to the emergency department. If advanced cardiac life support interventions are deemed unsuccessful, the patient is considered a potential organ donor. Exclusion criteria include unwitnessed arrests, a no-flow period exceeding 15 minutes, or a low-flow state longer than 60 minutes.

Upon clinical confirmation of death—defined as 5 minutes without any physical intervention—a 20-minute of nontouch period with electrocardiographic flatline is mandatory to declare the circulatory dead. After confirming death, an RM is performed (Table 4), and continuous positive airway pressure 10 cm H₂O with 100% FiO₂ with respiratory rate 10/min is maintained throughout the no-touch period. The consent of the next of kin is then requested.

**Table 4:** Recruitment Maneuver

PEEP 5 I/E ratio of 1:1 RR: 10 breaths per minute PC adjustments

Abbreviations: I/E, inspiratory-to-expiratory ratio; PC, pressure-controlled ventilation; PEEP, positive end-expiratory pressure; RR, respiratory rate.

After consent is obtained, heparin (10,000 UI IV) is administered, followed by 3 minutes of CPR, a second RM, and ventilation according to specific parameters (Table 5). If the chest radiograph reveals no abnormalities, the donor is transferred to the operating room.

**Table 5:** Respiratory Parameters

RR 4 breaths per minute VT 6 ml/kg PEEP 8 cm H₂O FiO₂ 100% I/E ratio 1:1

Abbreviations: I/E, inspiratory-to-expiratory ratio; PEEP, positive end-expiratory pressure; RR, respiratory rate; VT, tidal volume.

Bronchoscopy is performed to assess the bronchial content. Antegrade perfusion is initiated via the pulmonary artery using a fibrinolytic agent (15 mg recombinant tissue plasminogen activator), followed by Perfadex 60 ml/kg antegrade and 250 ml/vein retrograde) and then cold storage on ice. The lungs are subsequently transported for EVLP assessment and reconditioning.^14^, ^65^, ^66^

Healey et al^15^ published in 2020 the first North American experience with the uDCD lung-only using a lung protection strategy. After the cardiac arrest, the CPR was initiated, and the patient was transfer to the hospital. If the hospital team considered the resuscitation unsuccessful, the death is declared. When the family and coroner consent was obtained within 120 minutes from death, lungs were inflated with continuous positive airway pressure of 20 cm H_2_O and FiO_2_ of 50%. The donor is then moved to the operating room and ventilated with tidal volume 7 ml/kg, FiO_2_ of 50%, positive end-expiratory pressure of 5 cm H_2_O. Procurement is performed within 3 hours of death declaration. EVLP assessment and reconditioning are performed before transplantation acceptance.

## Results

The clinical experience with LTx from uDCD donors has been reported in the literature by 6 centers, covering a period from 2000 to 2019. Following Steen’s pivotal case report,^3^ larger case series have been published by the Spanish centers in Madrid^4^, ^47^, ^67^ and Santander,^13^ and later by Milan (Italy),^14^, ^65^, ^66^ and Toronto (Canada),^15^ each employing different procedural approaches, as previously described. Overall, more than 70 lung transplants from uDCD donors have been reported.

Across the centers included in the analysis, the number of potential uDCD lung donors ranged from 6 to 31 per study period. However, donor utilization rates varied widely, with only 23% to 50% of referred donors successfully proceeding to organ retrieval. This variability was influenced by factors such as initial donor condition, ischemic times, and preservation techniques but, more importantly, influenced by national legislative frameworks and by the inclusion/exclusion criteria applied by each institution(Table 6). Reported donor characteristics are limited; age and smoking history were relatively consistent across studies, with median donor ages between 39 and 50 years. Male donors predominated (92%-100%), and the proportion of donors with a history of smoking varied, reaching up to 54.4% in some cohorts (Table 7, Table 8).

**Table 6:** Criteria Differences Between Institutions

Center	Definition of WIT	Max.time of WIT	Definition of PT	Max. time of PT
H. of Lund (Lund)^3^	From declaration of death to topical cooling.	65 min	From topical cooling to EVLP	180 min
Puerta de Hierro (Madrid)^47^	From cardiac arrest to topical cooling	120 min	From topical cooling to procurement	240 min
Puerta de Hierro (Madrid)^4^	From cardiac arrest to topical cooling	150 min	From topical cooling to PA rinsing	240 min
H. 12 de Octubre (Madrid)^62^	From cardiac arrest to topical cooling	180 min	From topical cooling to PA procurement	240 min
H. Marqués de Valdecilla (Santander)^13^	From cardiac arrest to topical cooling	120 min	From topical cooling to PA procurement	180
Toronto General hospital (Toronto)^15^	From declaration of death to cold flush^a^	180 min	NA	NA
Policlinico H. (Milan)^65^	From cardiac arrest to pulmonary flush	-	From the end of CPR to reperfusion	-

Abbreviations: Max.time of PT, maximum time of PT allowed by protocol; Max.time of WIT, maximum time of WIT allowed by protocol; PT, preservation time; WIT, warm ischemic time.

a The maximum time from declaration of death to lung inflation was 120 minutes.

**Table 7:** Donor Characteristics

Center	Years of reported activity	Donors referred	Donors’ potential	Donors recovered	LT	Age (years)	Gender (M%)	Smoking history (%)	BMI	P/F
H. of Lund (Lund)^3^	2000	1	1	1	1	54	100	N/Av		
Puerta de Hierro (Madrid)^47^	01/2002-12/2012	N/Av	N/Av	N/Av	38	41.9 (14-55)	97.4	N/Av		
Puerta de Hierro (Madrid)^4^	01/2013-12/2019	215	31	17	14	50 (42-53)	92.9	54.4		461 (440-666)
H. 12 de Octubre (Madrid)^62^	06/2010-09/2011	15	6	4	3	39 (38-40)	N/Av	N/Av		345.3 (243-402)
H. Marqués de Valdecilla (Santander)^13^	10/2012-07/2018	22	9	7	8	41.5 (30-54)	100	N/Av		
Toronto General hospital (Toronto)^15^	02/2016-05/2019	147	44	16	5	54	100			
Policlinico H. (Milan)^65^	11/2014-07/2019	28	14	12	5	45 (20-57)	100	40	29.38 (24.49-34.6)	

Abbreviations: BMI, body mass index; LT, lung transplantation; P/F, oxygenation ratio.

**Table 8:** Recipient Characteristic

Center	Years of reported activity	Age (years)	Gender (M%)	BMI	Indication (%)	PH (%)	6MWT	LAS	Bridge (%)
H. of Lund (Lund)^3^	2000	54	0	17.72	COPD 100				0
Puerta de Hierro (Madrid)^47^	01/2002-12/2012	49.3 (24-66)	84.2	23.5 (±4.6)	COPD 42.1; Suppurative 28.9; IPF 21.1	13.2			
Puerta de Hierro (Madrid)^4^	01/2013-12/2019	55 (52-59)	92.9	26.4 (22-28)	COPD 50; Suppurative 14.3; IPF 21.4		329 (300-394)	33 (31.9-39)	0
H. 12 de Octubre (Madrid)^62^	06/2010-09/2011	57 (49-62)			COPD 100				
H. Marqués de Valdecilla (Santander)^13^	10/2012-07/2018	60.8 (58-67)	100		COPD 87.5; IPF 12.5				
Toronto General hospital (Toronto)^15^	02/2016-05/2019	54	0	17.72	COPD 100				0
Policlinico H. (Milan)^65^	11/2014-07/2019	52 (41-56)	100		COPD 40; IPF 60				20

Abbreviations: 6MWT, 6 m walking test; BMI, body mass index; COPD, chronic obstructive pulmonary disease; IPF, idiopatic pulmonary fibrosis; LAS, lung allocation score; PH, pulmonary hypertension.

Ischemic times are critical determinants of graft viability and post-transplant outcome. In the uDCD setting, this aspect is even more emphasized. Data collected across multiple centers highlight the variability in ischemic intervals. Total ischemic time (TIT) varied widely, with some centers—particularly Toronto and Milan-reporting values exceeding 1,000 minutes, Puerta de Hierro Hospital in Madrid reported TIT values of 657 ± 203 minutes for the first lung and 822 ± 225 minutes for the second lung in their earlier cohort. More recent data from the same center did not include TIT values but detailed WIT and preservation times.

WIT also exhibited substantial significant variability. Centers such as Puerta de Hierro and Hospital 12 de Octubre^62^ in Madrid reported WITs of approximately 120 to 150 minutes, whereas Santander documented shorter times, averaging 96.5 minutes; notably, the Milan cohort reported WITs of up to 250 minutes. Preservation and cold ischemic times further illustrate heterogeneity in donor management strategies. Centers employing early topical cooling and rapid PA flushing reported relatively shorter preservation times. In contrast, longer cold ischemic times suggest reliance on extended cold storage to manage recipient logistics (Table 9).

**Table 9:** Transplantation Characteristic

Center	Years of reported activity	TIT 1° lung (min)	TIT 2° lung (min)	WIT (min)	Preservation time/CIT (min)	CIT 1° lung (min)	CIT 2° lung (min)	CPB (%)	P/F	ECMO^a^	EVLP (%)
H. of Lund (Lund)^3^	2000			115	180	603		0		0	100
Puerta de Hierro (Madrid)^47^	01/2002-12/2012	657 ± 203	822 ± 225					31.6			21.1
Puerta de Hierro (Madrid)^4^	01/2013-12/2019			122.5 (83-150)	160 (110-235)	609 (585-720)^b^*	780 (720-810)	21.4	461 (440-666)	7.2	28.6
H. 12 de Octubre (Madrid)^62^	06/2010-09/2011		713 (675-760)	151.6 (140-165)					345.3 (243-402)		
H. Marqués de Valdecilla (Santander)^13^	10/2012-07/2018		678 (532-926)	96.5 (75-130)	169.7 (85-137)						14.2
Toronto General hospital (Toronto)^15^	02/2016-05/2019			115	180	603		0		0	100
Policlinico H. (Milan)^66^	11/2014-07/2019	1115		165.6 (106-199)							100

Abbreviations: CIT, cold ischemia time; CPB, cardiopulmonary bypass; EVLP, ex vivo lung perfusion; P/F, oxygenation ratio; TIT, total ischemia time; WIT, warm ischemia time.

a Use of intraoperative extracorporeal membrane oxygenation.

b PA flushing-recipient reperfusion.

Primary graft dysfunction (PGD) rates varied among studies, with grade 3 PGD incidence observed of 0% to 34.2%. Postoperative care requirements, including duration of mechanical ventilation and ICU stay, also differed. Median ICU stay ranged from 5 to 10.5 days, while overall hospital stay ranged from 17 to 35 days. Bronchial anastomotic complications, a topic of concern in uDCD transplantation, were reported in some series, with rates reaching 18.4%. However, it is important to distinguish between radiological findings and clinically significant complications, particularly given the small sample sizes (Table 10, Table 11).

**Table 10:** Short-Term Outcomes

Center	Years of reported activity	MV (days)	Tracheotomy (%)	NIMV (%)	ECLS (%)	PGD3-72 (%)	AR (%)	LI (%)	BAC (%)	ICU-LOS (days)	HLOS (days)
H. of Lund (Lund)^3^	2000	0	0	0	0					3	
Puerta de Hierro (Madrid)^47^	01/2002-12/2012	2.0 (0.5-98)	39.5	28.9	5.3		2.0 (0.5-98)	39.5	18.4	10.5 (1-120)	35.0 (1-128)
Puerta de Hierro (Madrid)^4^	01/2013-12/2019	3 (1-15)	42.9		21.4	21.3			7.1	9.5 (7-30)	45 (37-58)
H. 12 de Octubre (Madrid)^62^	06/2010-09/2011	2.3 (1-5)									56 (15-127)
H. Marqués de Valdecilla (Santander)^13^	10/2012-07/2018	2.5 (1-8.5)								9 (3-23)	
Toronto General hospital (Toronto)^15^	02/2016-05/2019	0	0	0	0					3	
Policlinico H. (Milan)^65^	11/2014-07/2019				20	0			18.2	5 (2-78)	17 (8-100)

Abbreviations: AR, acute rejection; BAC, bronchial anastomotic complications; ECLS, extracorporeal life support; HLOS, hospital days length of stay; ICU-LOS, intensive care unit length of stay; LI, lung infection; MV, mechanical ventilation; NIMV: noninvasive mechanical ventilation; PGD 3-72, primary graft dysfunction grade 3 at 72 hours.

**Table 11:** Long-Term Outcomes

Center	Years of reported activity	Best FEV1 (ml)	CLAD (%)	Freedom from CLAD 5 y (%)	Freedom from CLAD 10 y (%)	3 mo survival (%)	1 y survival (%)	3 y survival (%)	5 y survival (%)	10 y survival (%)
H. of Lund (Lund)^3^	2000					100				
Puerta de Hierro (Madrid)^47^	01/2002-12/2012			54	27	81.6	71.1	60.3	50.8	16.5
Puerta de Hierro (Madrid)^4^	01/2013-12/2019	2.8 (2.2-3.8)	7.1				85.7	85.7		
H. 12 de Octubre (Madrid)^62^	06/2010-09/2011									
H. Marqués de Valdecilla (Santander)^13^	10/2012-07/2018					87.5	87.5	87.5	87.5	
Toronto General hospital (Toronto)^15^	02/2016-05/2019					100				
Policlinico H. (Milan)^65^	11/2014-07/2019					100				

Abbreviation: CLAD, chronic lung allograft dysfunction.

Post-transplant survival outcomes were generally favorable. One-year survival rates ranged from 71% to 87.5%. Five-year survival reached 87.5% in some series, similar to outcomes observed with DBD lungs. Notably, freedom from chronic lung allograft dysfunction (CLAD) at 3 and 5 years was reported at 76.6% and 54%, respectively, with one study documenting a 10-year CLAD-free survival of 27% Studies comparing uDCD with DBD LTx indicate that, while early survival outcomes are similar, overall survival may be slightly lower for uDCD recipients. However, mid-term outcomes—including CLAD incidence and need for reintervention—are comparable. These findings underscore the need for further experience and expanded case series in this field (Table 11).

The cumulative experience with uDCD LTx demonstrates its viability as a strategy to expand the donor pool. Although logistical and technical barriers persist, improvements in preservation and evaluation—particularly EVLP—have led to excellent long-term outcomes, reinforcing the potential role of uDCD in routine clinical practice. While prolonged ischemia times have traditionally been associated with poor outcomes, advances in lung preservation techniques decrease these risks. Future efforts should focus on refining donor selection criteria and enhancing logistics to minimize ischemic injury while maximizing the utilization rates. Finally, new static preservation strategies using controlled temperatures also open possibilities for overcoming time constraints in this process.^68^

## The role of EVLP in UDCD lung programs

EVLP is a lung preservation strategy that enables the assessment and reconditioning of high-risk donor lungs.^69^ The clinical experience has demonstrated comparable outcomes between EVLP-treated lungs and standard donor lungs.^70^ Although EVLP is widely implemented and accepted in general LTx, its use in uDCD remains controversial. Some centers perform in vivo evaluation with favorable lung acceptance rates, while others prefer ex vivo assessment following EVLP.^15^, ^65^

Valdivia et al^47^ reported their experience with 38 uDCD lung transplants, including 8 assessed with EVLP between 2009 and 2012. They did not specify the number of donors accepted after in vivo versus EVLP evaluation. The incidence of PGD grade 2 to 3 was significantly higher in the uDCD group than in the DBD group (55.3% vs 33.2%, *p* < 0.008). Among the 8 uDCD lungs assessed with EVLP, 6 developed grade 2 to 3 PGD. However, no direct comparison was made between in vivo and EVLP groups. Higher mortality rates at 1, 3, 5, and 10 years were observed in the uDCD group compared to DBD, although EVLP-specific outcomes were not reported.

The same group reported an actuarial analysis covering 2013 to 2019.^4^ In vivo evaluation was the default strategy, with EVLP reserved for cases with concerns about lung viability. Only 14.4% of potential uDCD donors were offered, and 6.5% were ultimately transplanted. Lungs were accepted in 48.4% of cases following in vivo evaluation and 50% following EVLP (2 of 4). The incidence of PGD, postoperative ECMO requirement, and early complications was comparable between groups. No significant differences were found in short- or long-term survival among uDCD, cDCD, and DBD recipients. The group attributed improved outcomes to the replacement of cardiopulmonary bypass with intraoperative ECMO and emphasized that the availability of an EVLP platform was essential to the success of the uDCD program.

Suberviola et al^71^ reported their experience with 8 uDCD lung transplants between 2012 and 2018. They did not perform a comparative analysis between DBD and uDCD, but the outcomes were promising. The PGD 3 at 72 hours incidence was 25% and survival rate at 1 month, 1 year, 3 year, and 5 year was 100%, 87.5%, 87.5%, and 87.5%, respectively. EVLP was not used for the first 6 cases due to unavailability. After the EVLP program was implemented in 2017, the final 2 cases were evaluated ex vivo. The authors attributed their favorable results to short WITs and strict donor criteria but suggested that EVLP could enable extension of WIT in future cases.

Healy et al^15^ described the first North American experience with uDCD LTx in 2020. They performed EVLP evaluation in all cases. Among 30 consent donors, 14 were not recovered for medically unsuitable reasons, lack of recovery teams, or consent withdrawal. Of the 16 recovered, 2 were excluded pre-EVLP for medically unsuitable reasons. After EVLP, the ratio of acceptance was 35.7 (5 from 14). No PGD was observed, and 1-month survival was 100%; 4 of 5 patients were alive at a median of 651 days.

Palleschi et al^66^ compared outcomes from DBD and DCD donors (both cDCD and uDCD) from 2014 to 2019. Five of the donors were uDCD. From 14 uDCD approaches, 12 were recovered, and all were evaluated with EVLP. The ratio of acceptance was 41.6% (5 from 12). Although specific outcomes for the uDCD subgroup were not reported, overall DCD outcomes were comparable to those from DBD.

Most of the experience published was reported when the use of EVLP to validate and preserve the lung was still under investigation, and topical cooling and flush perfusion were the only described preservation method.^10^ Moreover, Hospital Puerta de Hierro emphasized the necessity of EVLP to support uDCD programs, and Santander transitioned to EVLP upon establishing their own platform.

While EVLP may not be mandatory in all uDCD cases, its availability appears critical to the success and expansion of uDCD lung transplant programs.

## Discussion

uDCD represents a promising but complex strategy for expanding the lung donor pool. This review highlights the physiological, technical, ethical, and legal considerations surrounding its implementation. Although clinical experiences remain relatively limited, the available evidence demonstrates the feasibility of using lungs from uDCD donors with acceptable short- and long-term outcomes. The reported experience of bithermia from 12 Octubre Hospital,^62^ the successful donation of lungs and kidneys from Santander group^13^ with hypothermic-non ventilated technique, the normothermic-oxygenated technique from Milan^66^ or Toronto,^15^ jointly with the knowledge of concomitant procurement of thoracic and abdominal organs in cDCD, could increase the uDCD programs self-sufficiency and cost-effectiveness obtaining abdominal and thoracic organs, when the legal and clinical framework allows it.

From a physiological standpoint, the lungs' unique ability to maintain cellular viability through alveolar oxygen diffusion, even in the absence of circulation, offers a critical and distinct advantage in the context of uDCD transplantation. Furthermore, the absence of the proinflammatory cascade associated with brain death may contribute to improved graft quality. Despite concerns regarding ischemia-reperfusion injury and mitochondrial dysfunction, emerging strategies such as EVLP and controlled ventilation during preservation phase have shown potential to mitigate these effects.

Clinical results from various centers demonstrate encouraging survival and graft function outcomes. However, variability in donor selection criteria, WIT, preservation strategies, and national legislative frameworks significantly impacts utilization rates and clinical success. The reported PGD rates and 1-year survival outcomes are comparable to those seen in standard DBD protocols, although some cohorts, the oldest one, have reported higher short-term complication rates, bronchial issues, and higher short- and long-term mortality. These findings underline the importance of robust lung assessment techniques, such as EVLP, before transplantation.

Ethical and legal complexities continue to be a major barrier to widespread implementation. Concerns regarding the definition of death based on circulatory criteria, the potential conflict between resuscitative efforts and organ preservation, and the risk of violating the “dead donor rule” during NRP require clear guidelines, transparency, and public trust. Additionally, the need for highly trained personnel and extensive logistical coordination renders uDCD programs resource-intensive, necessitating strong regional and institutional support.

## Conclusion

uDCD LTx is an evolving and increasingly viable approach to addressing the critical shortage of donor lungs. While the physiological characteristics of the lungs and advances in preservation techniques support its feasibility, numerous ethical, legal, and logistical challenges must be carefully addressed. Integrating uDCD into established transplant systems will require standardized clinical protocols, specialized team training, and targeted public education initiatives to foster trust and acceptance.

Future research should aim to optimize donor selection, minimize ischemic injury, and assess both the cost-effectiveness and long-term outcomes of uDCD protocols in larger, multicenter studies. With appropriate safeguards and continued innovation, uDCD has the potential to be a standard and effective component of LTx practice.

## Disclosure statement

This research did not receive any specific grant from funding agencies in the public, commercial, or not-for-profit sectors.

All the authors wrote, reviewed, and edited the manuscript.

## Declaration of Competing Interest

The authors declare that they have no known competing financial interests or personal relationships that could have appeared to influence the work reported in this paper.

## GLOSSARY

ΔΨm: mitochondrial membrane potential
CIT: cold ischemic time
CLAD: chronic lung allograft dysfunction
CPR: advanced cardiopulmonary resuscitation
DAMPs: damage-associated molecular patterns
DBD: donors after brain death
DCD: donation after circulatory death
cDCD: controlled donation after circulatory death
ECPR: extracorporeal cardiopulmonary resuscitation
ECMO: extracorporeal membrane oxygenation
EISOR: extracorporeal interval support for organ retrieval
EVLP: ex vivo lung perfusion
ETC: electron transport chain
FiO₂: fractional inspired oxygen
ICU: intensive care unit
LTx: Lung transplantation
mtDNA: mitochondrial DNA
NET: neutrophil extracellular trap
NRP: normothermic regional perfusion
PEEP: positive end-expiratory pressure
mPTP: mitochondrial permeability transition pore
PGD: Primary graft dysfunction
NHBD: Non-Heart Beating Donor
RM: recruitment maneuver
ROS: reactive oxygen species
TIT: Total ischemic time
uDCD: Uncontrolled donation after circulatory death
TV: tidal volume
WIT: warm ischemia time
